# Pilot case-control investigation of risk factors for hip fractures in the urban Indian population

**DOI:** 10.1186/1471-2474-11-49

**Published:** 2010-03-14

**Authors:** Ruchira M Jha, Ambrish Mithal, Nidhi Malhotra, Edward M Brown

**Affiliations:** 1 Harvard Medical School, Boston, MA 02115, USA; 2 Department of Endocrinology, Indraprastha Apollo Hospital, Sarita Vihar, New Delhi - 110 044, India; 3 Endocrine Division, Brigham and Women's Hospital, Boston, MA 02115, USA

## Abstract

**Background:**

Despite the reported high prevalence of osteoporosis in India, there have been no previous studies examining the risk factors for hip fracture in the Indian population.

**Methods:**

We carried out a case control investigation comprising 100 case subjects (57 women and 43 men) admitted with a first hip fracture into one of three hospitals across New Delhi. The 100 controls were age and sex matched subjects who were either healthy visitors not related to the case patients or hospital staff. Information from all subjects was obtained through a questionnaire based interview.

**Results:**

There was a significant increase in the number of cases of hip fracture with increasing age. There were significantly more women (57%) than men (43%). Univariate analysis identified protective effects for increased activity, exercise, calcium and vitamin supplements, almonds, fish, paneer (cottage cheese), curd (plain yogurt), and milk. However, tea and other caffeinated beverages were significant risk factors. In women, hormone/estrogen therapy appeared to have a marginal protective effect. For all cases, decreased agility, visual impairment, long term medications, chronic illnesses increased the risk of hip fracture. The multivariate analysis confirmed a protective effect of increased activity and also showed a decrease in hip fracture risk with increasing body mass index (odds ratio (OR) 0.024, 95% confidence interval (CI) 0.006-0.10 & OR 0.81, 95% CI 0.68-0.97 respectively). Individuals who take calcium supplements have a decreased risk of hip fracture (OR 0.076; CI 0.017-0.340), as do individuals who eat fish (OR 0.094; CI 0.020-0.431), and those who eat paneer (OR 0.152; 0.031-0.741). Tea drinkers have a higher risk of hip fracture (OR 22.8; 95% CI 3.73-139.43). Difficulty in getting up from a chair also appears to be an important risk factor for hip fractures (OR 14.53; 95% CI 3.86-54.23).

**Conclusions:**

In the urban Indian population, dietary calcium, vitamin D, increased body mass index, and higher activity levels have a significant protective effect on hip fracture. On the other hand, caffeine intake and decreased agility increase the risk of hip fracture. Future studies should be done in order to direct primary preventive programs for hip fracture in India.

## Background

Hip fractures are a major health problem in a developing country like India. They cause profound physical impairment, reduction in quality of life, admission to institutional care and also mortality especially in the elderly. The Indian population appears particularly vulnerable to the problem of osteoporosis and hip fractures[1]. It has been projected that by the next century 50% of all hip fractures in the world will occur in Asia. Current World Health Organization figures estimate that over 270 million people in India and China alone are likely to suffer from osteoporosis by the year 2020 [1-3]. Moreover, one of the only studies done on osteoporosis in India indicates that 29% of women and 24.3% of men between 20 and 79 years of age have a low bone mass index. This percentage increases to 50% in Indian women and 36% in Indian men over the age of 50[3].

Given these statistics, osteoporosis related increase in hip fractures poses a substantial burden both in terms of physical disability and health costs. Knowledge about the risk factors is essential for prevention of hip fractures. Risk factors for osteoporosis that have been described include female sex, low body mass index, old age, positive family history, early menopause or amenorrhoea, smoking, sedentary lifestyle, poor calcium intake and vitamin D deficiency [4-7]. Both calcium and vitamin D deficiencies are common in urban Indians [8-10].

Although the clinical manifestations of osteoporosis may be manifold, hip fractures are the most devastating consequence of this disease. It has been suggested that hip fractures occur at an earlier age in Indians in comparison with western Caucasian counterparts [1]. Although males are less at risk for developing osteoporosis [1], once they have developed the disease they appear to be at a greater risk of incurring a hip fracture [2]. However, there has been no previous study done on risk factors associated with hip fractures in Indian population.

The purpose of this pilot case-control investigation was to identify risk factors for hip fracture in an urban Indian population. The study incorporated risk and protective factors cited by previous research on other populations, and emphasized the importance of variables such as dietary calcium and vitamin D intake, and activity levels. These variables can potentially be targeted on a public health level.

## Methods

### Subject Selection

The study group consisted of 100 patients with a radiological confirmed diagnosis of a first hip fracture within the past *three *years. Persons with genetic collagen disorders (Osteogenesis Imperfecta) were excluded. The cases were admitted in 1 of 3 participating hospitals in New Delhi (Indraprastha Apollo Hospital, Max Hospital, and Sant Parmanand Hospital). Of the eligible, 55 men and 76 women contacted, 43 men (78.2%) and 57 women (75%) were available and willing to participate. The study was approved by the appropriate committees on human research, and all 100 patients included in the study provided informed consent.

The control group was recruited from healthy visitors (not related to the cases) and staff in the three hospitals; it consisted of 100 age (within 2 years) and sex matched individuals.

### Data Collection

The cases were interviewed in the outpatient clinic of the hospital in which they had been admitted. The controls were also interviewed in 1 of the 3 hospital settings. The following variables considered in other similar studies on risk factors for hip fracture, were determined for each subject (cases and controls) through the questionnaire- interview.

#### Basic Information

We ascertained the age, pulse rate, height (in inches) and weight (in kilograms) of all individuals.

#### Mode of Fracture

The cases were questioned as to how they had presented with hip fracture. They were asked whether they had a light fall, a heavy fall, other trauma such as an accident, or had merely felt pain and reported to their physician for subsequent diagnosis.

#### Visual Impairment

All subjects were asked whether or not they had glaucoma, cataracts or any other visual impairment. Those who wore spectacles or contact lenses but could see well with them were not classified as visually impaired.

#### Difficulty Getting up from a Chair

This was used as proxy for testing agility on both cases and controls. If the subject gave a history of not being able to get up from a chair effortlessly or without external assistance (prior to sustaining hip fracture), he or she was classified as positive.

#### Weight Loss

All subjects were questioned about whether or not they had lost a significant amount of body weight within the past one year {prior to sustaining hip fracture}.

#### Diet

Multiple items common in the Indian diet and noted as protective (such as those containing calcium or vitamin D) or risk factors (such as caffeine) for hip fracture were included in this section of the questionnaire. The subjects were asked whether they were vegetarian. They were also asked if within the past ten years they regularly drank tea, coffee, any other caffeinated drink, alcohol, and milk. If the subject responded in the affirmative to any of the above beverages, they were asked to quantify their intake as average number of glasses per day, week, or month. They were also questioned about smoking.

Similarly, all the subjects were questioned about their intake (and quantity per day, week, or month) of curd (plain yogurt), paneer (cottage cheese), fish, and almonds- the former two for their calcium content, and the latter two for an estimate of dietary vitamin D.

#### Calcium and Vitamin Supplements

Subjects were asked whether they had regularly taken calcium or vitamin supplements for a time period greater than one year within the past ten years. For the case subjects, this question specifically addressed a time before their hip fracture diagnosis and subsequent treatment. The frequency and duration of supplement intake was subsequently determined.

#### Prior Illness and Long term Medication

Information was obtained from all subjects on the presence and details of any major illnesses, prior injuries and hospitalizations, or any past surgeries. The frequency and duration (greater than 2 years) of any therapy was subsequently noted- specifically for common diseases such as hypertension, hypothyroidism, diabetes, asthma, cardiac problems, or whatever combinations existed. It was also recorded if the subject had been taking homeopathic or ayurvedic medication. Women were specifically asked if they had taken hormone/estrogen replacement therapy (HRT) for a time period greater than one year.

#### Activity and Exercise

The activity levels for all subjects were defined as 1 = bedridden, 2 = walks with the help of a stick or walker, 3 = walks without any support but leads a self-defined sedentary lifestyle, 4 = walks without any support and leads a self-defined active lifestyle.

All subjects were then asked whether or not they participated in any form of regular exercise in the past 5 years. This ranged from walking > 1 kilometer/day to strenuous workouts in a fitness center.

### Statistical Analysis

Univariate analyses were conducted to identify risk factors for hip fracture with logistic regression. The risk factors that were statistically significant based on a 5% significance level were used in a multivariate logistic regression model. A stepwise covariate selection algorithm was used to identify a set of risk factors that jointly affected the risk of hip fracture.

## Results

**Figure 1:**
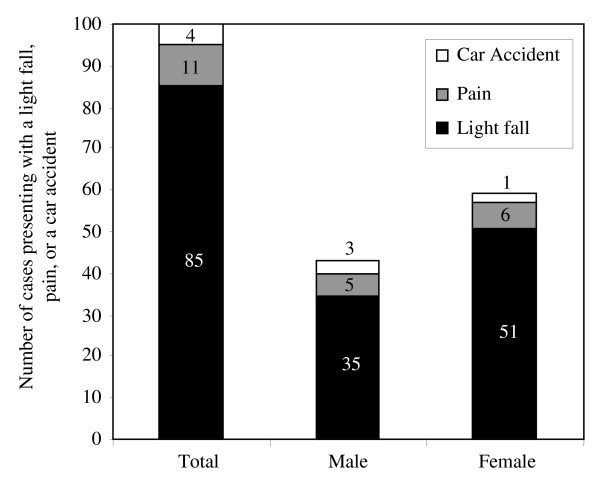
**Distribution of Fracture Presentation Modes**. In both men and women cases, most hip fractures were incurred due to a light fall. In some cases, merely pain was noted and hip fracture was diagnosed upon radiological evaluation. Motor vehicle accidents were not a major cause of hip fracture

General characteristics of both case and control subjects are shown in Table 1. As is evident from Figure 1, most case subjects presented after incurring a light fall (defined as slipping and falling on the ground, or a fall from a height of less than 2 feet).

**Table 1:** Characteristics of Cases and Controls

Variable	Case	Controls
Note: Plus - minus values are Means ± Standard Deviations	**(n = 100)**	**(n = 100)**
Sex Distribution:		
Male	43	43
Female	57	57

Mean Age	**65.7 ± 16.1**	**64.7 ± 13.8**
Male	69.9 ± 9.2	66 ± 4.2
Female	60.1 ± 20	63.1 ± 15.1

Mean Weight	**64 ± 10.4**	**67 ± 11.2**
Male	65.5 ± 12.5	68.6 ± 11.1
Female	62.8 ± 8.3	63.5 ± 10.3

Mean Height	**64.6 ± 4.7**	**65.9 ± 3.0**
Male	67.0 ± 5.8	67.4 ± 3.0
Female	62.9 ± 8.7	62.8 ± 2.8

Mean Body Mass Index (BMI)	**22.8 ± 3.8**	**23.8 ± 3.4**
Male	22.5 ± 3.1	23.3 ± 3.5
Female	24.8 ± 3.9	25.1 ± 5.1

% Taking Medication for:		
a) None	47	67
b) Hypertension (β-Blockers, Ace Inhibitors, Calcium Channel Blockers, Thiazide Diuretics)	27	15
c) Diabetes (Insulin, Sulfonylurea e.g. Glyburide, Biguanides eg. Metformin)	8	7
d) Asthma (β2 Agonists e.g. Epinephrine, Albuterol inhalers)	4	1
e) Cardiac	0	0
f) Homeopathy/Ayurveda	1	2
g) Cardiac & Hypertension on (Drugs listed in b, plus Aspirin, Nitroglycerin)	6	6
h) Diabetes & Hypertension (b and c)	3	2
i) Thyroid Hormone	4	0
Mean Pulse Rate:	75.9 ± 6.3	75.9 ± 6.3
Male	75.4 ± 6.4	75.4 ± 6.4
Female	76.4 ± 6.3	76.4 ± 6.3

**Figure 2:**
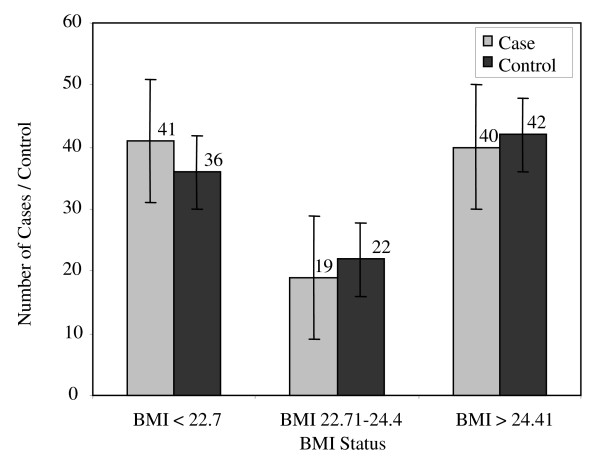
**Body Mass Index of 100 Cases and 100 Controls**.

**Figure 3:**
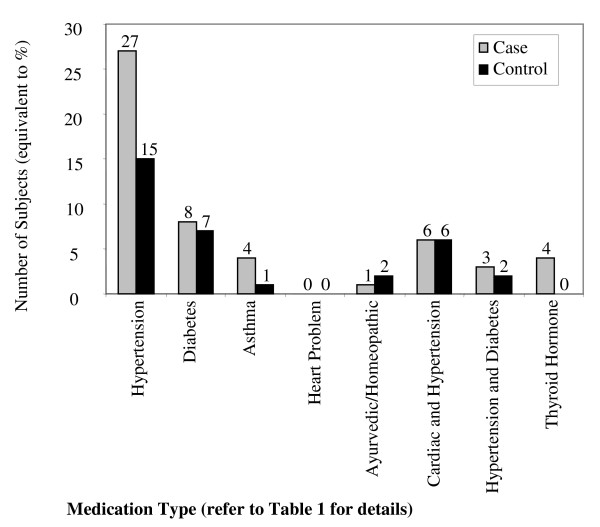
**Medications of 100 Cases and 100 Controls**. This illustration outlines the major medications taken by the 100 cases and 100 controls studied. More case patients were taking medication for hypertension and hypothyroidism as compared with their control counterparts. There was no such disparity between the two groups regarding the other medications listed.

**Figure 4:**
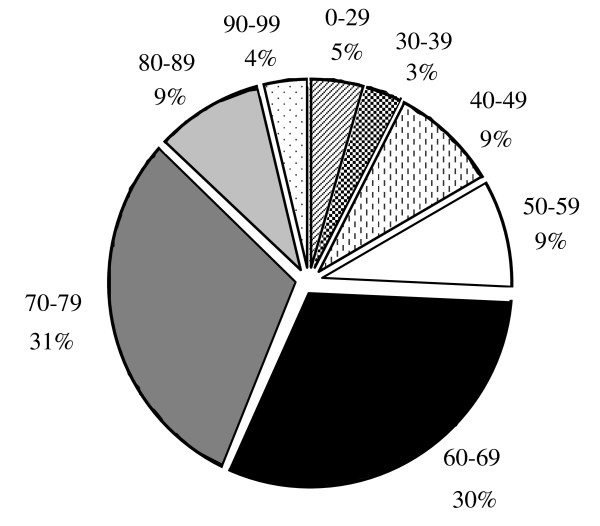
**Age Distribution of Hip Fractures**.

**Figure 5:**
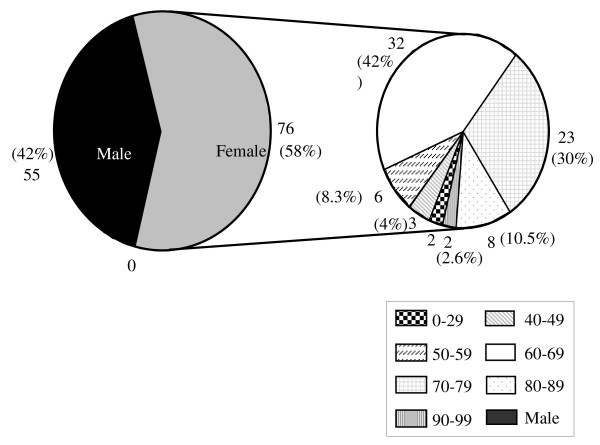
**Age Distribution by Sex**. (Shown for the Female Population)

**Figure 6:**
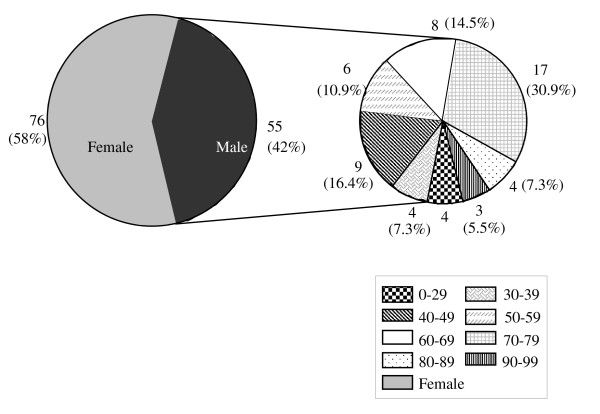
**Age Distribution by Sex**. Shown for the Male Population

There were no significant differences between the case patients and control subjects with regard to age. The weight and BMI of the controls were slightly larger as compared with the cases; however this difference was not statistically significant (Table 1, & Figure 2). Similarly more cases than controls appeared to be on long term medication for one of the chronic illnesses listed in Table 1 (Figure 3). The distribution of hip fractures between the sexes was interesting. Although only 57 women and 43 men were studied for all the risk factors, the information regarding sex was obtained using the initially eligible 55 men and 76 women. Thus, 58% of the fractures occurred in females and only 42% in males. Age between 60-79 years appears to be a major risk group with 61% of the hip fractures studied occurring in this age group (Figure 4). However, females within that age group seemed at a greater risk since 72% of all female hip fractures were between the ages of 60-79 as compared with 45.4% of the male fractures in the same age group (Figures 5 &6).

### Univariate Analysis

The odds ratios (OR) and 95% confidence intervals (CI) estimated by univariate logistic regression modeling for variables identified by previous research as possible risk or protective factors for hip fractures in other populations are presented in Table 2. The food items listed that contain calcium and vitamin D (such as milk, curd, paneer, almonds, and fish) or caffeine (such as tea) were those that are common in the Indian diet.

**Table 2:** Distribution and Logistic Regression Analysis of Selected Variables in Cases and Controls

	Variable	Cases (%) n = 100	Controls (%) n = 100	Odds Ratio (P-value; *95%*
1	Regular Exercise †	13 (13)	51 (51)	0.14 *(0.00; 0.07-0.29)*

2	Activity Level			
	• ≤ 2	80 (80)	17 (17)	1.00
	• > 2	20 (20)	83 (83)	0.05 *(0.00; 0.03-0.11)*

3	Take Vitamin Supplements ‡	14 (14)	46 (46)	0.19 *(0.00; 0.10-0.38)*
4	Take Calcium Supplements §	6 (6)	47 (47)	0.07 *(0.00; 0.03-0.18)*

5a	Eat Almonds **	19 (19)	44 (44)	0.30 (0.000; 0.16-0.56)
5b	Of those that eat Almonds, Quantity of			
	• ≤ 5 Once/day	4	3	1.00
	• > 5 Once/day	15 (78.9)	41 (93.7)	0.27 (0.01; 0.06-1.37)

6a	Eat Fish *	9 (9)	42 (42)	0.14 (0.00; 0.06-0.30)

6b	Of those that eat Fish, Quantity of Fish			
	• ≤ Once/week	4	6(14.29)	1.00
	• > Once/week	5	36 (85.7) † †	0.21 (0.03; 0.04-1.00)
7a	Eat Paneer (Cottage cheese) **	61 (61)	90 (90)	0.17 (0.000; 0.08-0.37)
7b	Of those that eat Paneer, Quantity			
	• ≤ 1 medium size serving Once/Week	28 (45.9)	27 (28.7)	1.00
	• > 1 medium size serving Once/Week	33 (54.1)	67 (72.3)	0.48 (0.03; 0.24-0.93)

8a	Eat Curd (Plain Yogurt) **	84 (84)	89 (89)	0.65 *(0.30; 0.29-1.48)*

8b	Of those that eat Curd, Quantity of Curd			
	• ≤ 2 cups/week	62 (76.5)	70 (71.4)	1.00
	• > 2 cups/week	19 (23.5)	28 (28.6)	0.77 (0.96; 0.39-1.51)
9	Drink Alcohol	15 (15)	19 (19)	0.75 *(0.36-1.58)*
10a	Milk intake**	49 (49)	59 (59)	0.67 *(0.16; 0.38-1.17)*
10b	Of those that drink Milk, Quantity of Milk			
	• ≤ 1 glass/day	39 (79.6)	32 (54)	1.00
	• > 1 glass/day	10 (20.4)	27 (45.8)	0.30 (0.006; 0.13-0.72)

11a	Regular Tea Drinkers**	9	8	2.11 *(0.07; 0.93-4.81)*

11b	Of those that drink Tea, Quantity of Tea			
	• ≤ 1 cup/day	14 (15.6)	18 (22.3)	1.0
	• > 1 cup/day	76 (84.4)	63 (77.7)	1.55 (0.26; 0.72-3.36)
12	Drink Other Caffeinated substances**	36 (36)	23 (23)	1.88 (0.05; 1.01-3.50)
13	Vegetarian	81 (81)	5	3.94 *(0.00; 2.09-7.43)*

14	Taken HRT for > 1 year	1	6 (10.53)	0.16 (0.09; 0.02-1.34)
15	Prior Injuries	2	6	0.32 *(0.17; .06-1.63)*
16	Prior Major‡‡	5	2	3.44 *(0.00; 1.91-6.22)*
17	On Long Term Medications §§***	5	3	2.09 *(0.01; 1.19-3.70)*
18	Difficulty getting up from a Chair (Agility)	7	2	12.60 *(0.00; 6.44-24.60)*
19	Eyesight difficulty	73 (73)	33 (33)	5.49 *(0.00; 2.99-10.07)*

† Within the past 5

‡ For a time period greater than 1 year within the past 10

§For a time period greater than 1 year within the past 10 Years

*Within the past 10

† Including coffee and carbonated caffeine drinks such as Coke and

‡ (Diabetes, Cardiac Disease, Hypertension, Asthma, Hypothyroidism, Any combination.

§Beta blockers, thiazide diuretics, ACE inhibitors, calcium channel blockers, insulin, sulfonylurea ((glyburide), biguanides (Metformin) b 2 Agonists e.g. Epinephrine, Albuterol inhalers, Aspirin, Nitroglycerine, Thyroid Hormone, Ayurvedic/Homeopathic medicine

#### General Physiology

The relative risk trends presented by this analysis are in accordance with those predicted by the previous literature. Patients with a visual impairment were more likely to have a hip fracture (OR 5.49; 95% CI 2.99-10.07), as were persons who had difficulty in getting up from a chair (OR 12.59; 95% CI 6.44-24.6), and vegetarians (OR 3.94; 95% CI 2.09-7.43).

#### Illnesses and Medications

Similarly persons with the chronic illnesses listed in Table 1 and those on long term medications were at a higher risk of hip fracture (OR 3.44; 95% CI 1.90-6.22 & OR 2.09; 95% CI 1.19-3.70 respectively). Although few women reported having taken HRT for more than 1 year, it appears that this therapy may have a protective effect (OR 0.16; p 0.091; 95% CI .018-1.34).

#### Caffeine

With regards to caffeine in the diet, both regular tea drinkers and persons who drank other caffeinated beverages were at a higher risk of hip fracture (OR 2.11; 95% CI .93-4.81 & OR 1.88; 95% CI 1.01-3.50 respectively). Although statistically not significant (p = 0.26), individuals who drank more than 1 cup of tea per day were 1.55 times more likely to have a hip fracture (95% CI 1.013-3.50). None of the individuals who drank alcohol consumed more than 1 glass a day- again this finding was not statistically significant. Considering the confidence interval, the effect of alcohol was ambiguous (OR 0.75; 95% CI .36-1.58).

Only three individuals (2 cases, 1 control, all men) gave a history of smoking. This was insufficient for any statistical analysis.

#### Calcium

All of the calcium containing food items considered in this study had a protective effect. Eating paneer reduced the risk of hip fracture by 82.6% (95% CI 0.081-0.374; p = 0.00). Moreover, if the subject ate paneer, eating more than 1 medium sized serving per week decreased the risk of hip fracture by 52.5% (95% CI = .242-.933; p = .03). Although not statistically significant, drinking milk reduced the risk of hip fracture by 43.2% (95% CI .38-1.16; p = .157), and eating curd reduced it by 35.1% (95% CI 0.29-1.48; p = .30). As expected, regular calcium supplements decreased the risk of hip fracture by 92.8% (OR 0.072; 95% CI 0.029-0.179; p = 0.00).

#### Vitamin D

Similarly, dietary vitamin D offers a protective role with regards to hip fracture. Not only did eating fish reduce the risk of hip fracture by 86.3% (95% CI 0.06-0.30; p = 0.00), but also individuals who ate fish more than once a week further decreased their risk of fracture by 79.2% (95% CI 0.04 - 1.0; p = 0.039). Almonds, another source of Vitamin D also reduced the risk of hip fracture (OR = 0.30; 95% CI 0.16-0.56; p = 0.00). Also, regular multivitamin supplements decreased the risk of hip fracture by 80.9% (95% CI 0.10-0.38; p = 0.00).

#### Activity

Another extremely important risk predictor appears to be activity levels and exercise. Regular exercise reduced the risk of hip fracture by 85.6% (95% CI 0.07-0.29; p = 0.00) and an activity level of greater than 2 decreased the risk by 94.9% (95% CI 0.03-0.11; p = 0.00).

### Multivariate Analysis

Table 3 presents the adjusted odds ratios, 95% confidence intervals, and p values of a multivariate logistic-regression model determined by stepwise selection. As expected in multivariate modeling, numerous risk factors had a reduced effect after adjustment for potentially correlated covariates. The factors that were statistically significantly associated with hip fracture were -activity levels, calcium intake (supplementary and dietary), fish intake (a measure of vitamin D), regular tea drinking (caffeine), difficulty getting up from a chair, age, and low BMI (general physiology). The former three variables (activity, calcium and fish intake) had protective effects on the risk of hip fracture, whereas the latter four variables were associated with an increased risk for hip fracture.

**Table 3:** Multivariate Analysis of Risk Factors for Hip Fractures

Variable	Adjusted Odds Ratio (95%C.I.)	P-value
Active Persons	0.024 (.006-0.100)	0.000
Take Calcium Supplements	0.076 (0.340-0.017)	0.001
Eat Paneer	0.152 (0.741-0.031	0.020
Eat Fish	0.094 (0.431-0.020)	0.002
Regular Tea Drinker	22.81 (3.730-139.4)	0.001
Difficulty getting up from Chair (Agility)	14.53 (3.860-54.23)	0.000
Age	0.920 (0.880-0.97)	0.000
Body Mass Index	0.81 (0.68-0.91)	0.021

Active persons (defined as those with activity level >2) have a 97.6% lower risk of hip fracture (95% CI 0.01-0.10). An increase of 1 in BMI resulted in an 18.6% reduced risk of hip fracture (95% CI 0.68-0.97). Decreased agility (difficulty in getting up from a chair) raised the chance of hip fracture (OR 14.5; 95% CI 3.87-54.23).

Regarding diet, calcium supplement intake reduced the risk of hip fracture by 92.4% (CI 0.017-0.340), those who ate paneer also had a lower chance of fracture (OR 0.152; CI 0.031-0.741) as did those who ate fish (OR 0.094; CU 0.020-0.431). Tea drinkers, on the other hand, had a greatly increased risk of hip fracture (OR 22.8; 95% CI 3.7-39.4).

## Discussion

Hip fracture is increasingly becoming a major health problem in India and knowledge about risk factors is essential for its prevention. Although many large scale studies have been reported in White and Asian (other than Indian) populations, there is currently no research available on variables that increase the risk of hip fracture in the Indian population. This pilot case control study revealed many vital risk factors for an urban Indian population. This section outlines the results of the study in the context of previous research, and indicates certain limitations of this study that should be focused on in future research.

Risk factors were assessed by interview through a standardized questionnaire. This analysis focused on identifying risk factors for hip fracture in the univariate and multivariate setting. A number of risk factors that were significant in the univariate analysis were not included in the final model produced by the stepwise selection routine. This is anticipated as the correlation between the risk factors may reduce their relative importance. In addition, effect modification may be present amongst the risk factors based on a comparison of odds ratios yielded by the univariate and multivariate models. For example, the effect of tea consumption is significantly elevated in the multivariate model. This suggests that the effect of tea consumption is modified by the presence of another risk factor in the multivariate model. Future investigations may include an in-depth analysis of interactions.

We found that dietary calcium, vitamin D, increased BMI, and higher activity levels have a protective effect on hip fractures. On the other hand caffeine intake (as measured by tea intake) and decreased agility increase the risk of hip fracture. Our results are largely in agreement with the studies conducted in White and Asian populations. In the Mediterranean osteoporosis study conducted in Europe, Johnell et al. found that late menarche, poor mental function, low BMI, lack of physical activity, low exposure to sunlight, and low consumption of calcium and tea to be significantly associated with the risk of hip fracture[11]. In the Asian osteoporosis study, Lau et al. found low dietary calcium intake, lack of physical activity, alcoholism and cigarette smoking to be risk factors for hip fracture[12]. Similarly, in Japan, Fujiwara et al found a low BMI, regular alcohol intake, prevalent vertebral fracture, having 5 or more children, a low milk intake and later age at menarche to be associated with risk of hip fracture[13].

Unlike the American and European population where the prevalence of hip fractures in women is more than twice that of men in any age group[6], the ratio in the Indian population appears to be 58% to 42%. Future research could explore this discrepancy. One possible reason could be the disparity in life expectancies in the different populations. The 2003 life expectancy at birth was 61.8 years for Indian males and 63.5 years for Indian females [14]. However the life expectancy at birth was 75.2 years for American males and 80.4 years for American females [15]. Thus not only are the life expectancies of Indians more balanced with regards to sex relative to the Americans, but the values are also significantly lower than their western counterparts.

Consistent with previous research [4,7,16,17], men and women with a lower BMI are at a significantly higher risk for hip fracture than their heavier counterparts. It has been suggested by these prior studies that this protection is a result of increased adipose tissue based production of estrogen, more padding around the hips that may decrease the energy transmission from the impact of the fall to the proximal femur, and the greater gravitational forces on bone mass[16,18]. However, this study did not examine the body distribution of adipose tissue. In American and European people, hip fracture risk increases with body height. However we did not find a significant association between risk of hip fracture and height.

Although not found significant in another research study[4] our data suggest that difficulty in getting up from a chair significantly raises the risk of hip fracture. It could be a risk factor for falls, but it could also be an important proxy for agility. More studies are required to determine the significance of this variable.

In accordance with previous research, physical activity greatly reduces the risk of hip fracture [12,16,19]. It is important however to quantify the load-bearing activity to determine its effect on bone mineral density.

Dietary calcium has been determined to be crucial in reducing the risk of hip fracture not only in this pilot study but also in other Asian studies involving the Chinese, Malaysian, Singaporean, Thai and Philippino populations[12,20]. A European study has shown that the risk of hip fracture increases with diminishing calcium intake in subjects whose daily intake was <500 mg. In the Asian study Lau et al. also found that diet calcium intake < 498 mg/d increases the risk of hip fracture. The multivariate analysis in this study indicated the importance of paneer (cottage cheese), a major component of the north Indian diet, and it also highlighted the importance of calcium supplements. Although calcium intake was quantified on a daily basis, future studies could examine the protective effect per gram of calcium. Given that dietary calcium intake in most Asian countries is low; calcium supplements should have a considerable impact on the reduction of hip fracture risk. Like physical activity, this variable is of great importance with respect to public health measures that can decrease the likelihood of hip fractures and thus relieve much of the morbidity and mortality associated with this condition in the Indian population.

Much like calcium, adequate vitamin D is essential for bone strength. Although the importance of almonds in the multivariate model was not apparent, intake of fish significantly decreases the risk of hip fracture in the population studied. Since vitamin D3 (with calcium) has been shown to reduce the risk of hip fractures in other elderly populations[21] and the fact that the Indian population is considered Vitamin D deficient[1,8,9], the effect of sunlight and other vitamin D containing foods should be examined for their effect on hip fracture incidence. Moreover, although some trials demonstrate that calcium and vitamin D supplementation is effective[21] others fail to indicate an effect of vitamin D alone [22,23]. Hypotheses regarding vitamin D deficiency in the urban Indian population include poor sunlight exposure, skin pigmentation, atmospheric pollution, and a vitamin D deficient diet[1,8]. Awumey et al. also reported altered vitamin D metabolism in cultured skin fibroblasts from Indians[24].

The effect of caffeine was estimated mainly by the consumption of tea- an extremely popular beverage with the Indian population. It is evident that increased intake of tea significantly increases the risk of hip fracture, however in order to get a more accurate estimate of the risk of caffeine, a future more nuanced analysis may be required to account for the protective effect of the milk within the tea. It is also important to separate subjects based on the quantity of milk in their tea as some individuals may enjoy black tea and others may like to add milk. In India, the majority prefer to add milk, albeit in varying amounts to their tea. Although previous research indicates that the effect of habitual tea drinking on bone density is minor and does not significantly alter the risk of fractures in an American population[25], this particular variable may be significant in the Indian population as 90% of the cases and 81% of the controls were regular tea drinkers.

A major limitation of the study is the relatively limited sample size which impacted the power to detect the effect of certain variables deemed relevant by previous literature such as hormone/estrogen therapy, thyroid hormone, alcohol consumption and smoking (there were only 3 smokers, all men). Other potential risk/protective factors were not assessed such as exposure to sunlight or bone mineral density.

Hormone/estrogen therapy had a protective effect on women when considered alone, however the power of the study to evaluate this effect in detail was limited due to the small sample size. Nonetheless, it is consistent with the hypotheses of other research studies that both recent and prolonged use of this therapy has a protective effect on bone density and thus decreases the risk of fractures[4]. Similarly, although 6 case members and 0 control members took thyroid hormone, the sample size was too small to estimate the effect of this drug on the risk of hip fracture. This is a potentially important variable as there is a current controversy about whether thyroid hormone may lead to osteoporosis and whether its use increases fracture risk at a clinical level[26].

This study indicated a potential inverse association with alcohol consumption and risk factor- again the sample size was inadequate to make a statistically significant determination. Previous research, like that of thyroid hormone, is controversial- some studies indicate that alcohol consumption weakly reduces risk of hip fracture[27], others suggest that there is no association between the two[5], and still others suggest that increased alcohol consumption increases the risk of hip fracture [4]. It may be difficult to distinguish a potential protective/risk effect of alcohol on the bone versus the increased risk of falling due to increased alcohol consumption.

It is also important to mention that as with all case-control investigations, there is a possibility of recall bias and it is not possible to validate the information provided by the subject. However, given these constraints, valuable information was retrieved from the analysis.

Unlike many previous studies that focused on women, this investigation included both males and females. The distribution of hip fracture by age and sex was found to be different from that in the western counterparts studied in other investigations[1,11,12]. As mentioned earlier, 50% of Indian women and 36% of Indian men over the age of 50 suffer from osteoporosis and are at risk for hip fracture. Notwithstanding the high prevalence of osteoporosis and related hip fractures in India, and the resulting morbidity and mortality associated with the condition, there is no prior existing research on the risk factors of hip fractures in the Indian population. Given this formidable public health problem, the results of this study may be beneficial on a national level. If further research substantiates the importance of these variables, increasing activity level, calcium and vitamin D intake, decreasing caffeine intake, and maintaining a healthy BMI may decrease the prevalence of hip fractures in the country. In addition to emphasizing these dietary and lifestyle improvements, public awareness programs can encourage early screening and treatment strategies. These would help to further reduce the prevalence and thus morbidity and mortality of hip fractures in India.

## Conclusion

In conclusion, this study has demonstrated that dietary calcium, vitamin D, increased body mass index, and higher activity levels have a significant protective effect on hip fracture in the urban north Indian population. On the other hand, caffeine intake and decreased agility increase the risk of hip fracture. Based on these findings, future studies should be done in order to direct primary preventive programs for hip fractures in India.

## Competing interests

The authors declare that they have no competing interests.

## Authors' contributions

RJ participated in the study design, acquisition of data, drafting of manuscript, AM participated in the study design, coordination, interpretation of data, drafting of the manuscript, and he gave final approval of the version to be published. NM participated in the interpretation of data, drafting of the manuscript and in revising it critically for important intellectual content. EB participated in study design, analysis and interpretation of data. All authors read and approved the final manuscript.

## Pre-publication history

The pre-publication history for this paper can be accessed here:

http://www.biomedcentral.com/1471-2474/11/49/prepub

## References

[B1] Malhotra N Mithal A Osteoporosis in Indians Indian J Med Res 2008 127 263 268 18497441

[B2] Gupta A Osteoporosis in India--the nutritional hypothesis Natl Med J India 1996 9 6 268 74 9111786

[B3] Pande KC Prevalence of low bone mass in healthy Indian population J Indian Med Assoc 2002 100 10 598 600 12452513

[B4] Grisso JA Kelsey JL Strom BL Risk factors for hip fracture in black women. The Northeast Hip Fracture Study Group N Engl J Med 1994 330 22 1555 9 10.1056/NEJM199406023302202 8177244

[B5] Cumming RG Klineberg RJ Case-control study of risk factors for hip fractures in the elderly Am J Epidemiol 1994 139 5 493 503 8154473 10.1093/oxfordjournals.aje.a117032

[B6] Cummings SR Melton LJ Epidemiology and outcomes of osteoporotic fractures Lancet 2002 359 9319 1761 7 10.1016/S0140-6736(02)08657-9 12049882

[B7] Kelsey JL Hoffman S Risk factors for hip fracture N Engl J Med 1987 316 7 404 6 3807977 10.1056/NEJM198702123160709

[B8] Arya V Bhambri R Mithal A Vitamin D status and its relationship with bone mineral density in healthy Asian Indians Osteoporos Int 2004 15 1 56 61 10.1007/s00198-003-1491-3 13680103

[B9] Goswami R Gupta N Goswami D Prevalence and significance of low 25-hydroxyvitamin D concentrations in healthy subjects in Delhi Am J Clin Nutr 2000 72 2 472 5 10919943 10.1093/ajcn/72.2.472

[B10] Tandon N Marwaha RK Kalra S Bone mineral parameters in healthy young Indian adults with optimal vitamin D availability Natl Med J India 2003 16 6 298 302 14765619

[B11] Johnell O Gullberg B Kanis JA Risk factors for hip fracture in European women: the MEDOS Study. Mediterranean Osteoporosis Study J Bone Miner Res 1995 10 11 1802 15 10.1002/jbmr.5650101125 8592959

[B12] Lau EM Suriwongpaisal P Lee JK Risk factors for hip fracture in Asian men and women: the Asian osteoporosis study J Bone Miner Res 2001 16 3 572 80 10.1359/jbmr.2001.16.3.572 11277276

[B13] Fujiwara S Epidemiology of osteoporosis in Japan J Bone Miner Metab 2005 23 Suppl 81 3 10.1007/BF03026329 15984420

[B14] Registrar General, India, SRS Based Abridged Life Tables 1999-2003 Statement 2 2006

[B15] Arias E National Vital Statistics Report - 2004 CDC 2007 56 9 18274319

[B16] Cummings SR Kelsey JL Nevitt MC Epidemiology of osteoporosis and osteoporotic fractures Epidemiol Rev 1985 7 178 208 3902494 10.1093/oxfordjournals.epirev.a036281

[B17] Pruzansky ME Turano M Luckey M Low body weight as a risk factor for hip fracture in both black and white women J Orthop Res 1989 7 2 192 7 10.1002/jor.1100070206 2918419

[B18] Cummings SR Nevitt MC A hypothesis: the causes of hip fractures J Gerontol 1989 44 4 M107 11 2738306 10.1093/geronj/44.4.m107

[B19] Norton R Galgali G Campbell AJ Is physical activity protective against hip fracture in frail older people? Age Ageing 2001 30 3 262 4 10.1093/ageing/30.3.262 11443030

[B20] Jitapunkul S Yuktananandana P Parkpian V Risk factors of hip fracture among Thai female patients J Med Assoc Thai 2001 84 11 1576 81 11853300

[B21] Chapuy MC Arlot ME Duboeuf F Vitamin D3 and calcium to prevent hip fractures in the elderly women N Engl J Med 1992 327 23 1637 42 1331788 10.1056/NEJM199212033272305

[B22] Lips P Graafmans WC Ooms ME Vitamin D supplementation and fracture incidence in elderly persons. A randomized, placebo-controlled clinical trial Ann Intern Med 1996 124 4 400 6 8554248 10.7326/0003-4819-124-4-199602150-00003

[B23] Cooper C Barker DJ Risk factors for hip fracture N Engl J Med 1995 332 12 814 5 10.1056/NEJM199503233321210 7862187

[B24] Awumey EM Mitra DA Hollis BW Vitamin D metabolism is altered in Asian Indians in the southern United States: a clinical research center study J Clin Endocrinol Metab 1998 83 1 169 73 10.1210/jc.83.1.169 9435436

[B25] Chen Z Pettinger MB Ritenbaugh C Habitual tea consumption and risk of osteoporosis: a prospective study in the women's health initiative observational cohort Am J Epidemiol 2003 158 8 772 81 10.1093/aje/kwg214 14561667

[B26] Eeden SK Van Den Barzilay JI Ettinger B Thyroid hormone use and the risk of hip fracture in women > or = 65 years: a case-control study J Womens Health (Larchmt) 2003 12 1 27 31 10.1089/154099903321154112 12639366

[B27] Baron JA Farahmand BY Weiderpass E Cigarette smoking, alcohol consumption, and risk of hip fracture in women Arch Intern Med 2001 161 7 983 8 10.1001/archinte.161.7.983 11295961

